# CT Findings in Pulmonary and Abdominal Sarcoidosis. Implications for Diagnosis and Classification

**DOI:** 10.3390/jcm9093028

**Published:** 2020-09-20

**Authors:** Claudio Tana, Iginio Donatiello, Maria Gabriella Coppola, Fabrizio Ricci, Marica Tina Maccarone, Tiziana Ciarambino, Francesco Cipollone, Maria Adele Giamberardino

**Affiliations:** 1 Geriatrics Clinic, “G. Bernabeo” Hospital, Contrada S. Liberata, 66026 Ortona (CH), Italy; 2 Internal Medicine Unit, University Hospital of Salerno, 84131 Salerno, Italy; iginiodonatiello@gmail.com; 3 Internal Medicine Unit, Ospedale del Mare, 80145 Napoli, Italy; gabry.cop@libero.it; 4 Department of Neuroscience, Imaging and Clinical Sciences, Institute of Advanced Biomedical Technologies, “G.d’Annunzio” University, 66100 Chieti, Italy; fabrizio.ricci@unich.it; 5 Imaging Department, Hospital of Pescara, 65123 Pescara, Italy; maccaronemarica@hotmail.com; 6 Internal Medicine Unit, Marcianise Hospital, 81025 Caserta, Italy; tiziana.ciarambino@gmail.com; 7 Medical Clinic, Department of Medicine and Science of Aging, “G. D’Annunzio”, University of Chieti, 66100 Chieti, Italy; fcipollone@unich.it; 8 Geriatrics Clinic, Department of Medicine and Science of Aging, “G. D’Annunzio” University of Chieti, 66100 Chieti, Italy; mag@unich.it

**Keywords:** sarcoidosis, computed tomography, imaging, biopsy, lungs, liver, spleen

## Abstract

Sarcoidosis is a granulomatous disorder of unknown etiology characterized by noncaseating granulomas virtually in every organ and tissue. This finding represents the most important diagnostic clue to reach a correct definition of sarcoidosis, although the biopsy is invasive and has several risk procedures. Several efforts are made to suspect the diagnosis of sarcoidosis by combining noninvasive elements, in particular from imaging, though these findings are often nonspecific and reflect the wide multifactorial pathogenesis. Every effort should be made to obtain a detailed radiological picture that, if associated with a suggestive clinical picture, could avoid the need of biopsy in some specific cases. In this narrative review, we aim to describe main computed tomography (CT) features of pulmonary and abdominal sarcoidosis, by reporting strengths and limits of this technique, in particular for the identification of extrapulmonary, isolated disease.

## 1. Introduction

Sarcoidosis is a granulomatous disorder of unknown etiology where noncaseating and nonnecrotizing granulomas represent the histopathological key of the disease [1]. Often, this finding represents the most important diagnostic clue to reach a correct definition of sarcoidosis, although the biopsy is invasive and has risk procedures such as infection and bleeding, depending on the site being investigated [2]. Several efforts are made to suspect the diagnosis of sarcoidosis by combining noninvasive elements, though these findings are often nonspecific and reflect the wide multifactorial pathogenesis. Hence, the importance of imaging methods is underlined where radiological findings are most clear, and every effort should be made to obtain a detailed radiological picture that, if associated with a suggestive clinical picture, could avoid the need of biopsy in some specific cases [3]. In this narrative review, we aim to describe main computed tomography (CT) features of pulmonary and abdominal sarcoidosis, by reporting strengths and limits of this technique, in particular for the identification of isolated extrapulmonary (abdominal) disease.

A literature search was performed on public databases (PubMed, Scopus). Search queries were the following: “sarcoidosis” AND imaging OR computed tomography; “sarcoid lesions” AND imaging OR computed tomography; “pulmonary” AND sarcoidosis OR sarcoid lesions; “lung” AND sarcoidosis OR sarcoid lesions; “abdominal” AND sarcoidosis OR sarcoid lesions. Duplicate papers were considered only once. Papers were excluded if the topic was not adherent to the theme of pulmonary, abdominal sarcoidosis and computed tomography. Papers with the main text not in English language were excluded as well.

## 2. Sarcoidosis, the Chameleon Disease

Sarcoidosis is a multifaceted disease where the clinical picture can be totally asymptomatic at the onset (in up to 50% of cases) [4] and clinical manifestations, when present, may be nonspecific and vary depending on the involved organs, and virtually any tissue can be affected. Since almost all organs can be affected by the disease, imaging techniques play an important role in the diagnosis of this disorder, and lung, abdominal sites, heart and brain are the main fields to detect the localization of the disease. Brain contrast-enhanced CT (CECT) and magnetic resonance imaging (MRI) of the heart can be useful to detect nodules and tissue enhancement (e.g., myocardium) in these districts.

The nonspecific presentation of several cases of sarcoidosis, in particular when there is no lung disease and the abdominal involvement is isolate, has led to the definition of chameleon disorder [4,5,6]. Lungs and intrathoracic lymph nodes are the sites most frequently involved (in up to 90% of cases), and the clinical picture ranges from totally asymptomatic, with only radiological findings, to overt restrictive lung disease [7,8].

At present, there is no agreement on the real incidence of gastrointestinal sarcoidosis, that is reported to be very rare (0.1% of all cases). However, the real epidemiology is unknown because most cases have an asymptomatic course, and it is supposed that the real incidence is larger than that actually described, as documented by postmortem studies [5,9].

Among all abdominal manifestations, the involvement of the liver and spleen is most frequent—reported to be around 10–25% of the cases. Various degrees of nonspecific liver test abnormalities can, however, be observed (increase in alanine and aspartate aminotransferase, gamma-gt, alkaline phosphatase), with or without constitutional symptoms such as weight loss, anorexia, fever and night sweats. Less frequently, symptoms of cholestasis such as pruritus and jaundice can be found [10,11].

## 3. CT in Pulmonary Sarcoidosis, Imaging Findings and Classification

**Figure 1:**
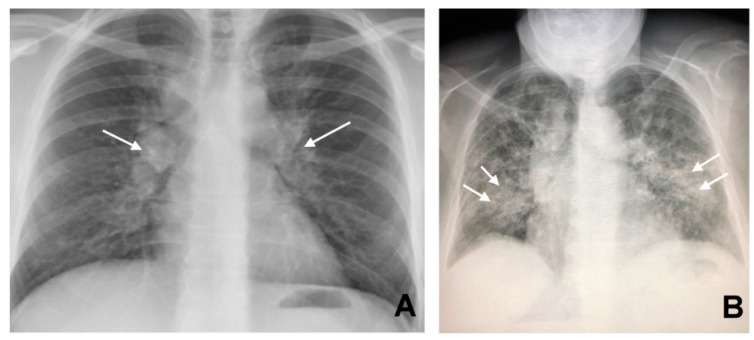
Bilateral hilar lymphadenopathy ((**A**) arrows) and bilateral parenchymal infiltrations with a tendency to confluence ((**B**) arrows).

**Figure 2:**
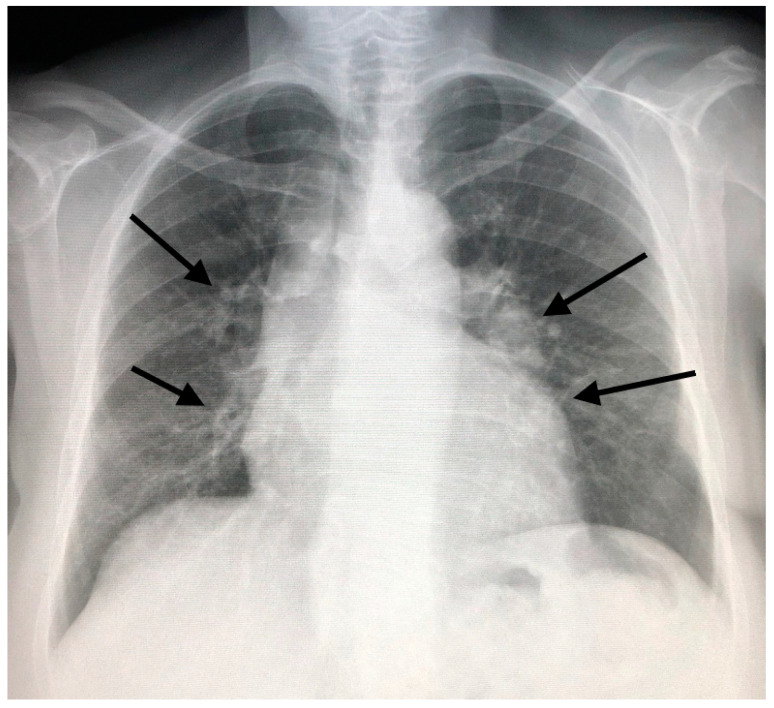
Bilateral pulmonary infiltrates and minimal mediastinal widening (arrows) in a patient with sarcoidosis.

In the past, before the diffusion of high-resolution CT (HRCT), which allows a clearer definition of the interstitium impairment from sarcoidosis, lung involvement patterns were defined based on the Scadding classification at chest X-ray. This classification defines as stage I the presence of bilateral hilar adenopathy (Figure 1, panel A), stage II bilateral hilar adenopathy and pulmonary infiltrates (Figure 1, panel B), stage III pulmonary infiltrates without overt hilar adenopathy (Figure 2), and stage IV the presence of overt pulmonary fibrosis [12]. Through HRCT examination, it has been shown that many radiological pictures classified as a 0–1 stage Scadding at X-rays presented even “more minimal” parenchymal alterations compatible with the disease.

**Figure 3:**
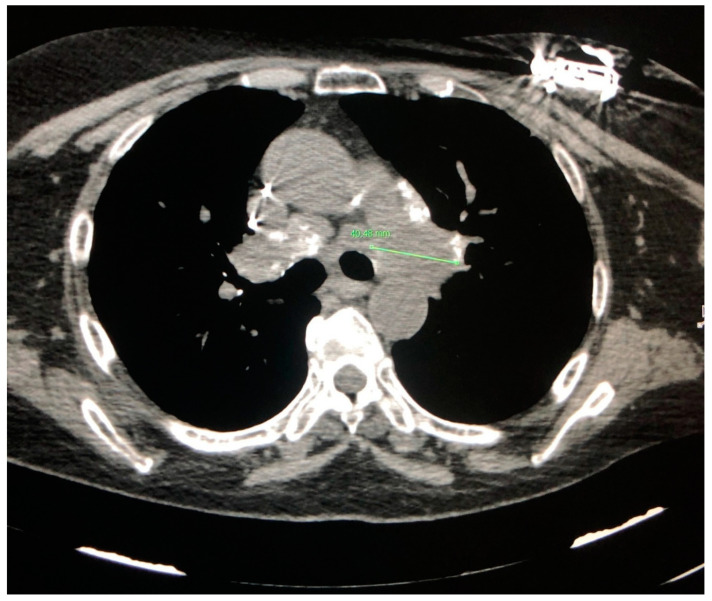
Subcarenal and hilar enlargement of lymph nodes with calcifications.

In an appropriate clinical context, there are also radiological HRCT patterns of mediastinal and parenchymal involvement which are virtually diagnostic of sarcoidosis. This is the case in which the enlargement of lymph nodes is present at the subcarenal level and symmetrically at the hilar level (Figure 3), associated with the presence of nodules with a typical perilymphatic distribution. These nodules, typically well defined, can have smooth or irregular margins and are commonly 2–5 mm in size. The most characteristic finding is, however, the coexistence of right paratracheal lymph node enlargement in addition to bilateral hilar enlargement (lambda pattern at scintigraphy and PET) [13,14].

**Figure 4:**
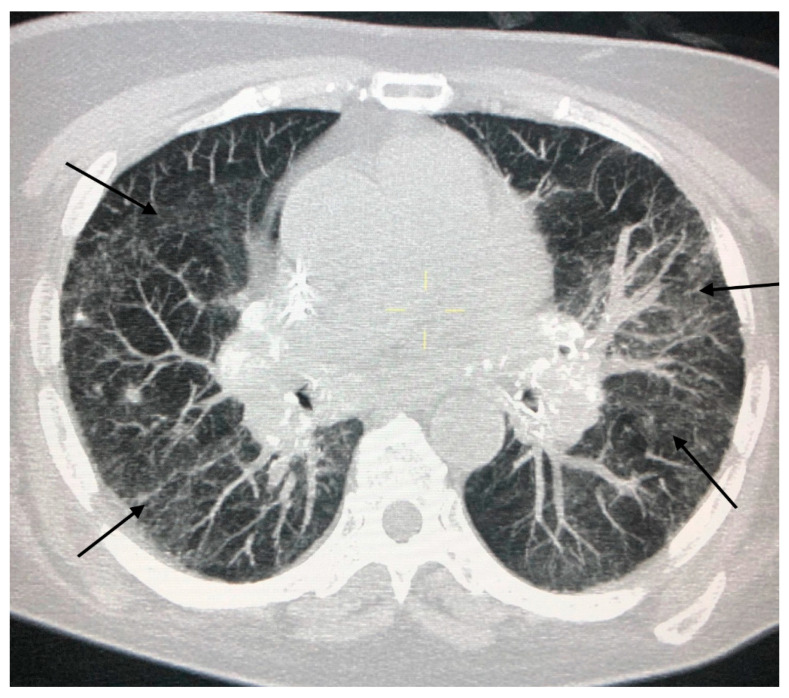
Bilateral parenchymal infiltrates that tend to merge into large pulmonary opacities (arrows).

Because of their typical lymphatic predilection, the nodules are mainly grouped along the bronchial vascular bundles, interlobular septa, interlobar fissures, and subpleural areas [15]. The nodules also affect the middle and upper lung fields and can merge to form larger opacities (Figure 4). The distribution of the granulomas (peribronchial and intraluminal) can explain the air trapping which reflects the involvement of the small airways, and can be detected more easily with expiratory HRCT scans. This regional air trapping is almost constant in all cases of sarcoidosis [16]. Consolidative and ground-glass lesions are frequently associated with the lesions described above. Calcifications of the nodules with “icing sugar” patterns constitute another characteristic finding of sarcoidosis, represented by the presence of amorphous calcifications within an enlarged lymph node [14]. The galaxy sign at HRCT is also highly suggestive of sarcoidosis and consists of a large nodule, usually with irregular margins, resulting from the coalescence of numerous smaller satellite nodules. Another relatively new finding at HRCT consists of the “sarcoid cluster sign”, described for the first time by Herraez Ortega, characterized by the grouping of small point nodules at the periphery of the lungs.

**Figure 5:**
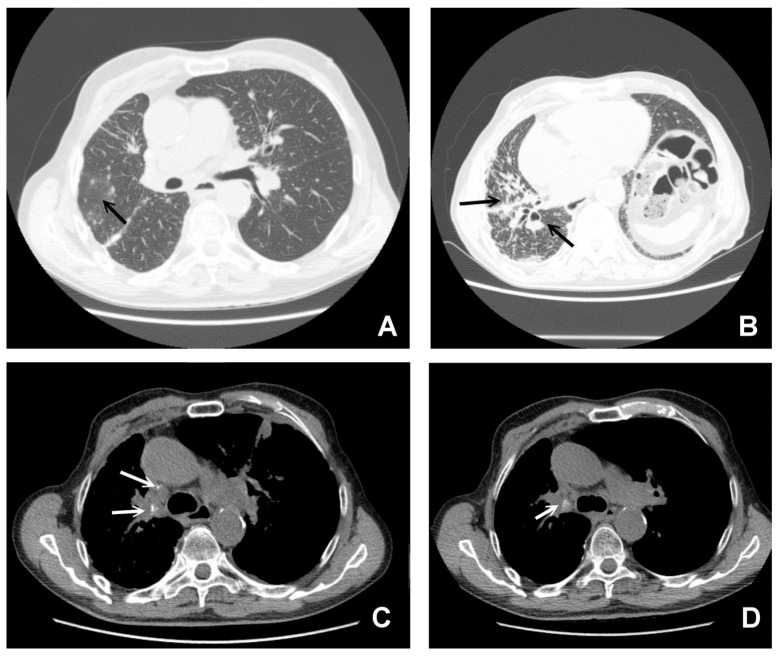
CT findings in pulmonary sarcoidosis. Panel (**A**) large nodule surrounded by numerous tiny satellite nodules (the “Galaxy sign”, arrow). Panel (**B**) shows multiple traction bronchiectasis with architectural distortion of the parenchyma (black arrows). Panel (**C**,**D**) (white arrows) shows the presence of calcifications of enlarged lymph nodes.

In addition to presenting with typical radiological characteristics, sarcoidosis can manifest with a wide spectrum of atypical HRCT images such as unilateral and asymmetric hilar or mediastinal lymphadenopathy of the parenchyma that mimic a mass, ground-glass opacity, miliary distribution of opacities, thickening of the interlobar septa and fibrocystic changes [13,14,17]. Figure 5 shows some CT features of pulmonary sarcoidosis. Even if chest HRCT has several advantages in terms of the detection of nodules that are not otherwise evident on chest x-rays, sometimes it is too detailed and can show a plethora of patterns that could be misdiagnosed with those observed in other disorders if not analyzed correctly [13].

Atypical findings at HRCT raise several issues of differential diagnosis with other primitive or secondary lung disorders. Lymphangitic carcinomatosis (LC) differs from sarcoidosis because here the nodules are more distributed along the fissures and in the subpleural area compared to those of LC [18]. Furthermore, the septal thickening is inconstantly present, less homogeneous and never prevalent as compared to LC. The prevalent distribution to the upper lobes and the absence of pleural effusion and peribronchial cuffing also orient toward the sarcoidoisis diagnosis.

When sarcoid nodules are isolated, more voluminous and confluent in the subpleural area, they can mimic the pseudo-plates of silicosis. Additionally, the presence of fibrotic lesions confluent in parailar conglomerates, and the distribution of lymph node calcifications with “eggshell” patterns are radiological findings that can mimic silicosis [12]. The key to a correct diagnosis of sarcoidosis is the predominant nodular and mid–upper craniocaudal distribution of the lesions.

When lymph node involvement from sarcoidosis is more asymmetric and unilateral, especially if it is associated with less typical consolidative lung lesions, the suspicion is of a lymphoproliferative disorder, which in some forms can also be associated with peribroncovascular nodules; the predominant nodular and mid–upper craniocaudal perilymphatic distribution of the lesions can orient toward sarcoidosis, as stated before.

A disorder that can be misdiagnosed as sarcoidosis and that manifests similarly to lymphoma is the multicentric Castleman disease (MCD). This rare disease affects lymph nodes and related tissues diffusely and causes fever, lymph node enlargement, dyspnea, hepato and splenomegaly, weight loss and loss of appetite. Typical findings are the lymph node enlargement found at CECT and, unlike sarcoidosis, the serum elevation of IL-6 levels. Biopsy is mandatory to reach the definitive diagnosis [18]. Similar CECT features of sarcoidosis can also be found in the IgG4-related diseases, which are a group of disorders characterized by lymphocytes, IgG4-secreting plasma-cells and fibrous infiltration of the tissues, and clinically by retroperitoneal fibrosis, autoimmune pancreatitis, eye disease and enlargement of lacrimal and salivary glands. As in sarcoidosis, there is a rapid response to steroids [13]. Some interstitial fibrotic disorders such as the chronic form of Hypersensitivity Pneumonia and Idiopathic Pulmonary Fibrosis (IPF) can be characterized by the same distribution of fibrotic and nodular lesions. Here, the association with typical lymph node calcifications can suggest the diagnosis of sarcoidosis [18,19,20]. Among interstitial disorders, also connective tissue diseases (CTD)-related ILD, such as that from Sjogren syndrome, enter into the differential diagnosis. Generally, they are characterized by a predominant reticular pattern with traction bronchiectasis and early signs of honeycombing, with basilar predominance in the NSIP pattern. Ground-glass opacities have often a symmetric distribution involving mostly middle and lower lung fields [19,20].

Sarcoidosis can sometimes affect the small airways and manifest with airflow obstruction, mimicking COPD. Additionally, lung carcinoma can be erroneously misdiagnosed as sarcoidosis, in particular when the irregular nodular lesions are single and located unilaterally [18].

Tuberculosis (TB) should always enter into differential diagnosis with sarcoidosis, especially in the high-risk areas considered and in immunocompromised patients. More specific characteristics of TB reactivation are the HRCT presence of exudative lesions, acinar nodules and the tree-in-bud sign. Lymph node calcifications from TB can be recognized because they are denser than in sarcoidosis and tend to be more often unilateral, while in sarcoidosis, they are more often bilateral [21].

The problems become more challenging when the two disorders coexist; in this case, the need for a biopsy is high to reach the correct diagnosis, in particular when sarcoidosis has an atypical clinical course [21,22].

**Figure 6:**
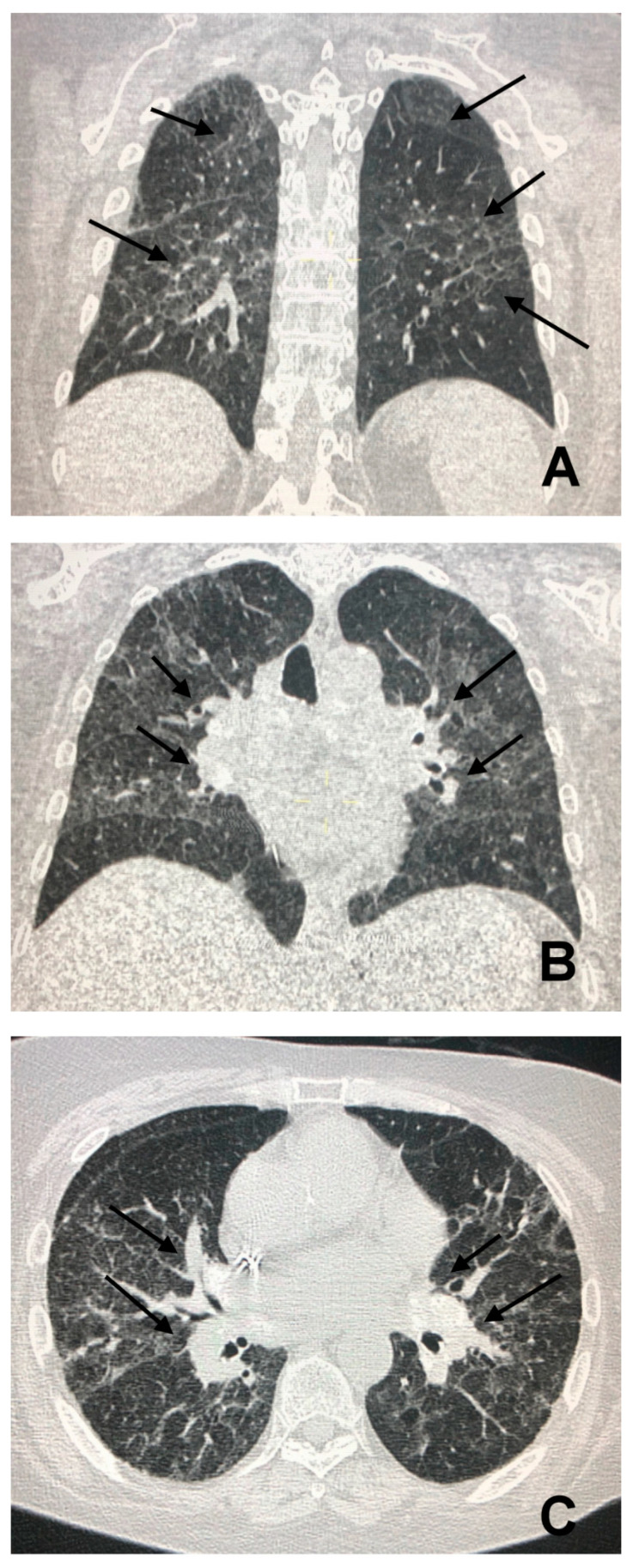
Panel (**A**) shows bilateral and asymmetrical coarse opacities of both pulmonary fields (arrows). In Panel (**B**), parenchymal infiltrates are associated with hilar enlargement (arrows), also evident in Panel (**C**) (arrows).

In the case of a patient’s presentation in the full fibrotic phase, the diagnosis of sarcoidosis can be suggested by the characteristic centrifugal and dorsolateral distribution of fibrotic lesions with a prevalence in the upper lung fields. In advanced stages, the characteristics of fibrosis can include linear opacities, pulmonary arch distortion, traction bronchiectasis and honeycombing located mainly in the upper lung fields (Figure 6) [23].

HRCT can be indirectly helpful to evaluate the prognosis of sarcoidosis patients by differentiating reversible from irreversible lesions. Lesions such as micronodules, nodules or peribroncovascular thickenings can undergo regression spontaneously or after treatment. Ground glass lesions have variable reversibility. Irreversible lesions include honeycombing, traction bronchiectasis and emphysematous bubbles [24]. Several HRCT scores have been developed to correlate the imaging manifestations with the results of respiratory function tests in pulmonary sarcoidosis [25,26].

A recent HRCT score based on the presence of ground glass opacities, thickening of the interlobular septa and parenchymal consolidations can allow the quantification of the disease activity and predict the variation of forced vital capacity after a year of therapy [25,26]. Walsh et al. reported that both the visual assessment of the extent of pulmonary fibrosis and the relationship between the diameter of the pulmonary artery and the diameter of the ascending aorta on HRCT can provide useful information about the prognosis of sarcoidosis.

Another integrated clinical and radiological staging system combines a composite physiological index (derived from the diffusion capacity of carbohydrate monoxide (TLCO or DLCO), forced vital capacity and forced expiratory volume in the 1st second of expiration, FEV_1_) with characteristic images at HRCT (e.g., presence and extension of fibrosis, ground glass opacities, emphysema and traction bronchiectasis). This score has demonstrated a high predictive value in terms of mortality [26].

HRCT can also be useful in sarcoidosis to reveal some complications such as mycetomas that occur in 2% of all patients with sarcoidosis and have a higher incidence in patients with pulmonary fibrosis, pre-existing lung cavities, cystic lung disease and bronchiectasis. Mycetomas are masses within a cavity in the fibrotic forms, that can be a potential source of fatal hemoptysis [27]. Another possible complication in pulmonary sarcoidosis is the development of pulmonary hypertension. CT is more sensitive than chest X-ray to reveal signs of pulmonary hypertension and can highlight both dilatation of the pulmonary trunk exceeding 29 mm in diameter and dilatation of the right and left pulmonary arteries [17]. The ratio of the diameter of the main pulmonary artery to the diameter of the ascending aorta has been demonstrated to be an excellent predictor of mortality regardless of the other CT patterns when it tends to be greater than 1, in the context of CPI <= 40 [26].

### The Integration of 18-Fluoro-Deoxyglucose (FDG) Positron Emission Computed Tomography (PET/CT)

18-FDG PET/CT has an important role in the evaluation of the inflammatory activity in several disorders, infectious, inflammatory and not. Recently, some studies have found that 18-FDG PET/CT (more than 67Ga scintigraphy) can also be accurate to reveal the presence of hyperactivity from sarcoid lesions in each involved organ, and can detect and quantify well the active inflammation of granulomatous lung lesions [28,29]. Some 18-FDG PET/CT patterns can be observed, such as a thick linear FDG uptake, called the “tiger man” sign [30,31], and four stages can be distinguished on the basis of the presence and extent of organ involvement: type I, thoracic lymph nodes; II, lung parenchyma; type III, peripheral lymph nodes; and type IV, system organ involvement. The evaluation of disease activity is a very valuable tool in these cases of doubts concerning the activity of lesions (fibrosis vs. active inflammation) and when the treatment initiation or continuation/discontinuation is considered [32].

It should be considered that the positive scan should be interpreted with caution because the FDG uptake can also be present in other inflammatory processes and neoplastic diseases. We believe that the usefulness of 18-FDG-PET can be reserved mostly for the staging and follow-up of sarcoidosis, and also for evaluating the effect of the treatment, because most lesions can “turn off” at scans of follow-up after a successfully therapy [31,33].

## 4. CT Features of Abdominal Sarcoidosis

### 4.1. The Involvement of Liver, Spleen and Abdominal Lymph Nodes

**Figure 7:**
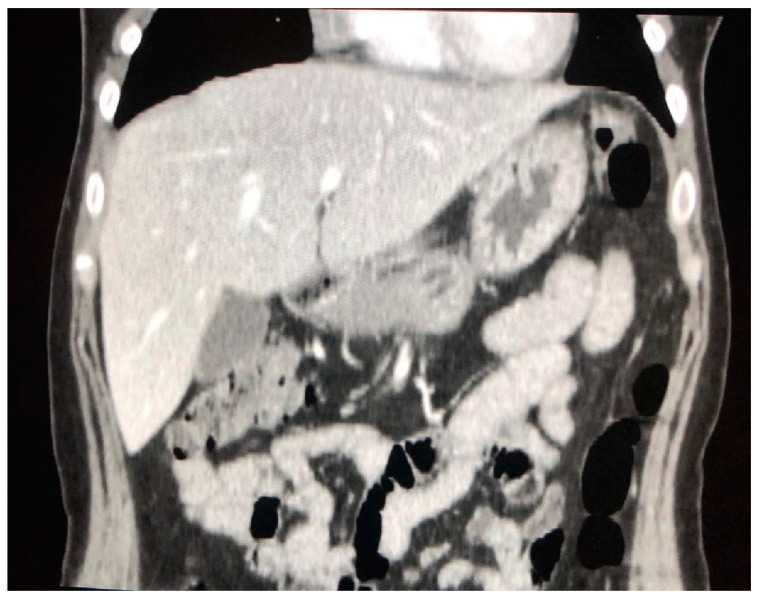
Massive hepatomegaly in a patient with sarcoidosis.

Liver is the abdominal site most often affected with sarcoidosis, but its involvement is often underestimated, due to a pauci or asymptomatic course, while it can be found in up to 50–79% of patients at biopsy and 67–70% in autopsies [34,35]. Beyond the nonspecific constitutional symptoms mentioned before, the hepatic damage can manifest less frequently with overt liver dysfunction, hepatic cirrhosis or portal hypertension and is usually associated with the formation of hepatic granulomas [36,37]. Granulomas promote a chronic fibroblastic reaction with consequent onset of fibrous septa within the periportal regions and consequent transformation in cirrhosis and portal hypertension [38]. CT can show nonspecific imaging features such as organomegaly (Figure 7), abdominal lymph node enlargement in celiac region and at hepatic hilum [35,39]. In some cases, the liver parenchyma is not homogeneous and shows the presence of low-density intrahepatic septa at CECT. An analysis of Warshauer et al. reported an incidence of 29% of liver size greater than 20 cm at CECT, and of 8% of a marked hepatic enlargement (liver size > 25 cm). Their analysis found only a 5% nodule frequency within the liver, but other analyses showed a different incidence ranging from 0 to 19%, that can be attributed in part to a different slicing capability of the used scanners [40,41].

Liver nodules appear usually as hypodense, nonenhancing lesions of variable size on CECT images (from 1–3 mm to several centimeters) [35,39]. Singular lesions are observed as large solitary and hypovascular masses of low density, with or without intrahepatic bile duct dilatation or as multiple smaller low density, even hypovascular lesions [41].

The presence of focal calcifications is uncommon, and usually manifests as hyperdense, homogeneous areas, round or oval in shape on CECT [41]. Typically, the cortisone therapy reduces the number and size of hepatic lesions, usually after 3–5 months; therefore, CECT is useful not only for diagnostic purposes but also for follow-up of lesions over time, and to ascertain the success of the treatment [42].

**Figure 8:**
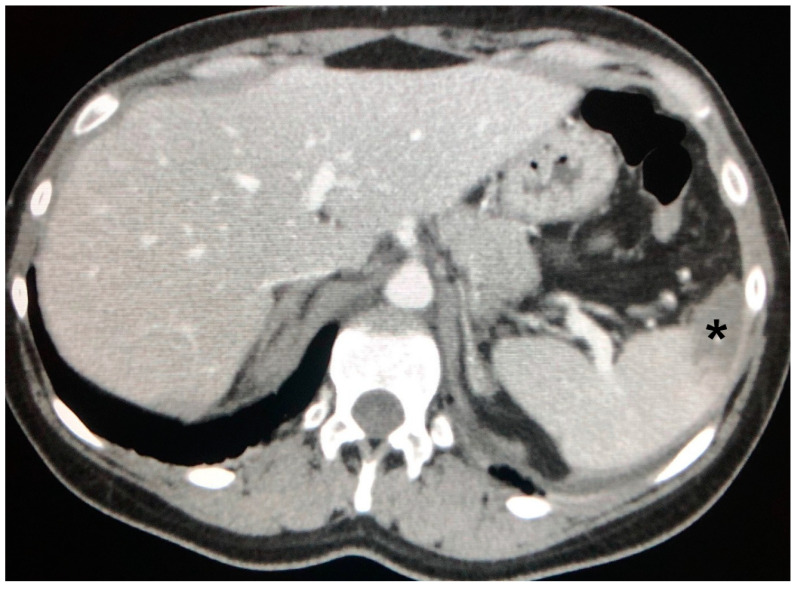
Splenic enlargement in a case of sarcoidosis, here with the presence of overt infarction (asterisk).

Liver is the organ that is less frequently involved alone in sarcoidosis; indeed, it is often associated with splenic disease [41]. Folz et al. observed that 75% of patients with hepatic sarcoidosis had also splenic and lymph node involvement [41], with an average incidence of splenic disease of about 40%, which is represented mostly by organ enlargement (Figure 8) [43].

Splenic nodules can be observed in up to 15% of abdominal CECT, and are seen as single or multiple hypodense lesions larger than 1 cm, with an irregular shape and a confluence tendency. Punctate, hyperdense calcifications have been found in up to 16% of the patients, and also more clearly calcified lesions have been reported [41,42].

The differential diagnosis of isolated liver or splenic nodules includes tuberculosis, primitive benign and malignant tumors, lymphoma, metastasis, vascular lesions such as hemangioma and hematoma and infectious diseases, such as abscesses. A biopsy attempt should be considered for a definitive diagnosis in patients without typical clinical and radiological features of sarcoidosis [39].

**Figure 9:**
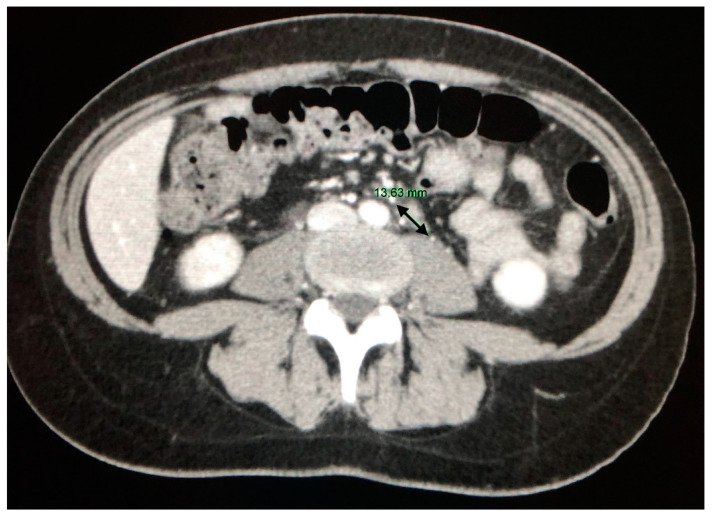
Lymph nodes enlargement with a maximum size of 13.63 mm (arrow) in the para-aortic site.

Abdominal lymph node enlargement is present in about 30% of patients and is mainly located in the hepatic hilum, para-aortic (Figure 9), celiac sites, around iliac vessels or in the mesentery. Lesions usually appear hypodense, and their size ranges from 1 to 2 cm. Lymph nodes greater than 2 cm are observed in up to 10% of patients, and raise problems of differential diagnosis with malignant lesions such as lymphoma [40,41] The study of Britt et al. confirmed that lymph nodes in sarcoidosis are basically smaller, less confluent, unlike non-Hodgkin lymphomas (NHL), that are most frequently larger (mean size of 8 ± 5.5 cm in NHL versus 2.6 ±1.7 cm in sarcoidosis, *p* < 0.01) and more often located in the retrocrural area [44].

In recent years, contrast-enhanced ultrasound (CEUS) has demonstrated to be a novel and promising method in the imaging of liver and spleen sarcoidosis. Ultrasound contrast agents (UCAs) are safe and do not interfere with the kidney function. CEUS can reveal parenchymal inhomogeneity when baseline US is negative. Furthermore, hepatic lesions that are hypoechoic at baseline US, show, after UCA injection, a variable arterial enhancement and progressive hypoenhancement in the portal-venous and late phases. Additionally, hypoechoic splenic lesions appear as progressive hypoenhancing nodules compared to the adjacent splenic tissue, in both the arterial and parenchymal phases [10].

### 4.2. Gastrointestinal Tract, Peritoneum, and Pancreas

The gastrointestinal (GI) tract is rarely affected with sarcoidosis, about 10% of all manifestations; every part of the GI tract can be involved. It is usually asymptomatic and often associated with lung disease [34].

The gastric antrum is the most frequently affected area. The granulomatous infiltration of the antral mucosa can be localized with ulcer formation, gastritis or nodular irregularities that are similar to polyps or can be diffuse, producing a thickening of the mucosa and a reduction in the lumen. Radiological findings can range from a peptic ulcerlike appearance to a clear thickening of the mucosa that mimics the Menetrier’s precancerous disease or gastric cancer [34,45,46].

Colon sarcoidosis is even rarer, and can be asymptomatic or manifest less frequently with symptoms of intestinal obstruction such as constipation or abdominal pain [44]. Imaging and endoscopic findings are not specifically diagnostic for sarcoidosis and can mimic other diseases, such as chronic inflammatory bowel disease; infectious diseases, e.g., tuberculosis and fungal infection; lymphoma and carcinoma [9,47].

Rare cases of appendicular sarcoid involvement have been described in the literature in association with the systemic disease. In granulomatous appendicitis, CECT shows an appendage of significantly increased dimensions and soft tissue density, absence of periappendiceal fluid and distinguishable walls, unlike acute appendicitis [48].

The peritoneal involvement is extremely rare in sarcoidosis and can manifest as exudative ascites and single or multiple granulomatous nodules in the peritoneum. CT is an excellent method to evaluate the peritoneum and can allow the identification of peritoneal sarcoidosis, that is characterized by ascites and/or peritoneal thickening as well as hypoattenuating nodular infiltrates of the peritoneal ligaments and mesentery [49].

The differential diagnosis of peritoneal sarcoidosis includes ovarian cancer, carcinomatosis, eosinophilic gastroenteritis, amyloidosis, typical infections such as tuberculosis, Whipple disease and fungal infections. Some small granulomatous nodules are not visible on the abdomen CT, and therefore, laparotomy and laparoscopy are indicated to evaluate the involvement of the parietal and visceral peritoneum [34,50,51].

Enlarged mesenteric and abdominal lymph nodes are the most common cause of intestinal obstruction due to extrinsic compression [34,39]. Pancreatic sarcoidosis is uncommon (1–3% of cases) and rarely symptomatic, most often mimicking isolated masses of the pancreas such as carcinoma. In the literature, pancreatic sarcoidosis has been described either as a pancreatic mass usually in the head of the pancreas or as a diffusely indurated nodular disease. Symptoms related to the presence of pancreatic sarcoidosis, if present, are caused by infiltration and inflammation of the pancreatic tissue or by dilatation of the common bile duct and pancreatic duct mimicking pancreatitis or pancreatic cancer. On CT scans, pancreatic sarcoidosis manifests as solitary pancreatic masses even 6–7 cm large, completely indistinguishable from pancreatic cancer, which are hypodense and hypo- or nonenhancing in association with dilatation of the biliary tree or peripancreatic lymphadenopathy. The absence of other clinical findings for systemic sarcoidosis requires surgical resection of the isolated mass to obtain the specific diagnosis [52,53].

### 4.3. Renal Involvement from Sarcoidosis

The exact incidence of renal sarcoidosis remains unclear, because sometimes image findings are absent and patients can have only laboratory alterations of the calcium metabolism. However, it is hypothesized to be rare and, when present, renal sarcoidosis has various manifestations on imaging. The granulomatous glomerulonephritis from direct infiltration is uncommon and the renal function is usually preserved [54]. The presence of interstitial nephritis can manifest at CECT with the typical striated nephrogram, represented by hypodense lines in the renal parenchyma [34,39]. The granulomatous renal pseudotumors are rare, and the appearance of these lesions on imaging is nonspecific. The described masses may have various features such as exophytic and nondeforming lesions. These masses can be hypo-, iso-, or hyperattenuating lesions on unenhanced CT and often are hypoenhancing on CECT [55,56]. 18-FDG-PET/CT can show a diffuse or patchy FDG uptake in kidney [57]. Carcinoma, metastases, lymphoma and oncocytoma are included in the differential diagnosis of such renal nodules [55,56].

## 5. Clinical Issues about the Extrapulmonary Involvement and Integrated Approaches for the Diagnosis of Sarcoidosis

Since the first joint statement by the American Thoracic Society (ATS), European Respiratory Society (ERS) and the World Association of Sarcoidosis and Other Granulomatous Disorders (WASOG) in 1999, the diagnostic criteria of sarcoidosis have remained almost unchanged and are based on the finding of a suggestive clinical picture, evidence of non-necrotizing granulomas at biopsy and exclusion of alternative causes of granulomatous inflammation [58]. This definition has been largely accepted over time and is useful to identify most of the cases, in particular of lung involvement, but does not resolve many problems of disease identification, especially when sarcoidosis has an extrapulmonary diffusion and an atypical presentation at the onset.

In the past, the Case Control Etiology of Sarcoidosis Study (ACCESS) Sarcoidosis Organ Assessment Instrument has been employed for more than ten years to evaluate the probability of organ involvement from sarcoidosis [59].

However, the ACCESS instrument failed to identify all possible organs affected with sarcoidosis, becoming outdated over time. A new instrument has, therefore, been developed. In the WASOG Sarcoidosis Organ Assessment Instrument, clinical experts on Sarcoidosis were asked to define the probability of sarcoidosis organ involvement by using a Delphy study methodology, and an expert agreement of at least 70% was needed to obtain the consensus.

Clinical manifestations were assessed as highly probable, when there was a likelihood of sarcoidosis causing the manifestation of at least 90%; probable, with a likelihood of sarcoidosis of between 50 and 90%; possible, with a likelihood of sarcoidosis of less than 50%; and indeterminate when there was no consensus [60]. Although more accurate than the ACCESS, also the WASOG instrument assumed that the patient had a known history of sarcoidosis to evaluate the probability of a specific organ involvement. The diagnostic issue, therefore, remains if there is not a certain diagnosis of sarcoidosis and when there is a singular organ involvement without typical lung disease and clinical presentation, which can raise even the slightest suspicion of the disease.

Recently, the diffusion of machine learning, a novel and promising technique that allows computers to learn and analyze imaging patterns like humans, is increasing the capacity of detection and interpretation of findings, quality of postprocessing and of reporting imaging features. This could translate into an improvement of diagnostic accuracy of traditional imaging techniques, and can result in an increase in operator performance [61]. In sarcoidosis, this method could be useful to detect early imaging findings that are otherwise not visible except with a high level of expert knowledge, and might contribute to reduce the risk of misdiagnosis and bad interpretation. Novel algorithms could be designed to define the probability of sarcoidosis with higher levels of accuracy than those that are available at present [62].

### 5.1. Suspected Pulmonary Sarcoidosis

An integrated clinical, laboratory and imaging investigation could provide a thorough approach to the patient with suspected sarcoidosis. In particular, clinical pictures that are highly suspicious, such as Lofgren’s, Heerfordt’s syndrome and Lupus pernio, are supportive of probable sarcoidosis. In the past, several efforts have been made to evaluate the usefulness of the Angiotensin Converting Enzyme (ACE) for the diagnosis of sarcoidosis, but several studies found a very low test accuracy and concluded that ACE levels are neither sensitive nor specific, as they can also be found to be elevated in other granulomatous disorders. Their elevation could not, therefore, be considered as diagnostic of sarcoidosis. [63]. ACE levels above 50% of the upper normal limit are indeed considered abnormal but cannot be sufficient to provide a suspicion of sarcoidosis; other tests are thus needed. Recently, it has been demonstrated that the soluble interleukin 2 receptor (sIL-2R) has higher sensitivity and specificity than ACE in establishing the diagnosis of sarcoidosis, and can be useful before the biopsy [64]. An elevation of serum calcium and, more frequently, the presence of hypercalciuria, indicates an altered vitamin D metabolism and is characteristic of sarcoidosis. Serum calcium should be dosed and monitored in all patients, as altered levels can lead to severe kidney disease and pancreatitis over time if not adequately treated [65].

Bronchoalveolar lavage (BAL) could reveal a significant lymphocytosis or an elevated CD4:CD8 ratio (>3.5), but is not sufficient, alone, to provide the specific diagnosis either of sarcoidosis or of any interstitial disease, though it can be helpful to reveal a suggestive pattern of eosinophilic or hypersensitivity pneumonitis and also to exclude tumors or infectious disorders [66,67]. Several lung disorders enter indeed into the differential diagnosis, such as lymphangitic carcinomatosis, lymphoma, bronchiolitis, tuberculosis, fungal infections, hypersensitivity, usual interstitial and cryptogenic organizing pneumonia and Langerhans cell histiocytosis [67]. Despite the low diagnostic accuracy of BAL for sarcoidosis, the presence of increased CD4/CD8 >3.5, in addition to a typical radiological picture and clinical context, is a feature specific enough to justify refraining from a biopsy. Briefly, the presence of imaging features that are highly suggestive of pulmonary sarcoidosis, such as bilateral hilar adenopathy if associated with the typical clinical pictures mentioned above, does not require biopsy and is sufficient to provide the diagnosis, as recommended by the recent American Thoracic Society (ATS) guidelines on sarcoidosis [65]. For less typical findings such as upper lobe or diffuse infiltrates, peribronchial thickening and enlarged extra thoracic nodes, the biopsy is mandatory to reveal (or exclude) the presence of typical noncaseating granulomas and to support alternative causes [65].

### 5.2. Screening for Extrapulmonary (Abdominal) Disease

While it is easier to suspect the presence of extrapulmonary involvement in patients who have an established diagnosis of sarcoidosis [68], the problem becomes more complex when organs are individually involved and lesions are found incidentally on imaging. Fortunately, these cases are very rare, and often require the biopsy for a definitive diagnosis to be established [69,70]. In patients with established pulmonary sarcoidosis, recent evidence from a systematic review demonstrated that liver tests abnormalities can be found in up to 12% of patients. Although the dysfunction of liver tests does not seem to follow a characteristic pattern, it has been found that an increase in alkaline phosphatase (less frequently transaminases) is associated with a higher prevalence of liver granulomas. There was no improvement, however, of liver tests after treatment, although the real therapy effectiveness could have been underestimated due to multiple confounding factors such as the association of transaminitis with the prednisone treatment in many cases. Furthermore, the research was not designed to evaluate the therapy outcome; therefore, a putative effect may exist. More research is needed, but a baseline testing could be recommended of serum alkaline phosphatase in sarcoidosis patients without an established hepatic disease, in order to screen the liver function [66].

In kidney disease, the renal impairment can be caused by three main mechanisms, namely the altered vitamin D metabolism, that can result from hypercalcemia and hypercalciuria mainly due to the overproduction of 1,25-dihydroxyvitamin by mononuclear cells trapped in pulmonary alveoli, and consequent nephrolithiasis and calcinosis; the granulomatous inflammation of the parenchyma, generally without progressive reduction in glomerular function up to renal failure; and acute interstitial nephritis with or without formation of granulomas [65]. The alteration of calcium metabolism is most often the cause of renal failure, followed by the acute interstitial granulomatous nephritis. From the recent metanalysis of Crouser et al., a renal dysfunction was found only in 7% of the selected studies, with a higher frequency of granulomas and nephrocalcinosis (ranging from 1 to 63% and 0 to 50%, respectively). Despite this low prevalence, in line with the recent ATS guidelines we recommend a routine laboratory test of renal function in patients with sarcoidosis, to rule out the presence of kidney impairment mainly because of renal disease, which is often asymptomatic, is associated with a poor prognosis. An early treatment could, therefore, be effective as most patients respond to the therapy in terms of clinical and laboratory improvement [65,71,72].

## 6. Conclusions

Although the diagnosis of pulmonary and abdominal sarcoidosis is often insidious, CT represents a valid diagnostic aid that can lead to the definitive diagnosis in many cases, especially in the presence of characteristic clinical pictures, such as Lofgren’s, Heerfordt’s syndrome and Lupus pernio. Here, the association with some findings such as bilateral hilar adenopathy is enough to achieve the diagnosis. Histological examination remains the gold standard to reach the definitive diagnosis, especially when radiological findings are atypical or found in isolation. In abdominal sarcoidosis, since the diagnosis is more challenging because baseline US could be falsely negative and CECT not indicated as a screening tool, it could be interesting to assess the diagnostic accuracy of novel methods such as CEUS as compared to CECT. The aim would be to evaluate which method reflects the best cost–benefit ratio to routinely detect nodules in sarcoidosis patients. New applications of machine learning could, in the future, increase the capacity of detection and imaging analysis in patients with sarcoidosis, but more studies are needed before their use in clinical practice.

Finally, the promising role of PET/CT opens the way for future studies of the disease and for more applications in the follow-up, for both pulmonary and abdominal manifestations of sarcoidosis.
